# What’s Coming Near? The Influence of Dynamical Visual Stimuli on Nociceptive Processing

**DOI:** 10.1371/journal.pone.0155864

**Published:** 2016-05-25

**Authors:** Annick L. De Paepe, Geert Crombez, Valéry Legrain

**Affiliations:** 1 Department of Experimental-Clinical and Health Psychology, Ghent University, Ghent, Belgium; 2 Institute of Neuroscience, Université catholique de Louvain, Brussels Woluwe, Belgium; 3 Centre for Pain Research, University of Bath, Bath, United Kingdom; Centre de Neuroscience Cognitive, FRANCE

## Abstract

Objects approaching us may pose a threat, and signal the need to initiate defensive behavior. Detecting these objects early is crucial to either avoid the object or prepare for contact most efficiently. This requires the construction of a coherent representation of our body, and the space closely surrounding our body, i.e. the peripersonal space. This study, with 27 healthy volunteers, investigated how the processing of nociceptive stimuli applied to the hand is influenced by dynamical visual stimuli either approaching or receding from the hand. On each trial a visual stimulus was either approaching or receding the participant’s left or right hand. At different temporal delays from the onset of the visual stimulus, a nociceptive stimulus was applied either at the same or the opposite hand, so that it was presented when the visual stimulus was perceived at varying distances from the hand. Participants were asked to respond as fast as possible at which side they perceived a nociceptive stimulus. We found that reaction times were fastest when the visual stimulus appeared near the stimulated hand. Moreover, investigating the influence of the visual stimuli along the continuous spatial range (from near to far) showed that approaching lights had a stronger spatially dependent effect on nociceptive processing, compared to receding lights. These results suggest that the coding of nociceptive information in a peripersonal frame of reference may constitute a safety margin around the body that is designed to protect it from potential physical threat.

## 1. Introduction

Localizing potentially harmful objects approaching our body is essential to adequately defend ourselves [1,2]. This ability requires the construction of a coherent representation of our body, and the space closely surrounding our body, i.e. the peripersonal space. The peripersonal space serves as a multisensory motor interface between our body and the environment [3,4], in which information from the body surface (e.g. tactile or nociceptive stimuli) is integrated with information from the external world (e.g. visual or auditory stimuli). This enables us to interact with the world: we can reach and grasp objects, and we can also avoid objects or defend ourselves against threatening objects intruding our peripersonal space. In monkeys this ability has been found to rely on bimodal visuotactile neurons in the ventral premotor cortex and the ventral intraparietal sulcus [5], which fire both for tactile stimuli and for visual stimuli presented near the stimulated area. Similarly, Dong et al. [6] found neurons in area 7b of the inferior parietal lobe of monkeys, that respond to nociceptive stimuli and to dynamical visual stimuli moving towards the receptive fields of these neurons. Dong et al. [6] suggested that this area provides visuo-somatic information about potentially noxious stimuli, and that it directs motor adjustments so that body exposure and contact with the threatening stimuli is minimized. In humans, a similar system has been proposed for tactile and visual stimuli (for a review, see [7]), and more recently also for nociceptive and visual stimuli [8–12]. However, unlike animal studies, most of the behavioral research in humans has used external (e.g. visual) stimuli at only two fixed locations (i.e. one position near the participants, and one far from the participants), instead of dynamical stimuli. There are several reasons why it could be more interesting to study the influence of *dynamical* stimuli on nociceptive (and tactile) processing. First, it would increase the ecological validity of the studies, as in real life objects are continuously moving around in the environment. Second, it would make research in humans more comparable to the animal studies mentioned above investigating multisensory integration within the peripersonal space [5,6]. Third, the neural systems representing the peripersonal space show a preference for moving stimuli over static stimuli, both in monkeys and in humans. In monkeys, visual and tactile responses of some of the bimodal neurons in the premotor cortex are directionally specific [13–15]. Moreover, the firing rates of some of these neurons change dynamically with stimulus velocity [14]. Also in humans there is some evidence that approaching visual, auditory and tactile stimuli evoke increased neural activity within the intraparietal sulcus and the ventral premotor cortex [16,17]. Because of the relevance of moving objects to the peripersonal space system, Canzoneri, Magosso, & Serino [18] developed a paradigm enabling to investigate the influence of dynamical *auditory* stimuli on *tactile* processing. In this task, Canzoneri et al. [18] measured reaction times (RTs) to a tactile stimulus applied to the right index finger while dynamical sounds, which gave the impression of either approaching or receding from the subject’s hand, were presented. Tactile stimulation was delivered at different temporal delays from the onset of the sound, such that it occurred when the sound source was perceived at varying distances from the body. Participants were asked to respond as fast as possible, trying to ignore the sound. They found that an auditory stimulus speeded up the processing of a tactile stimulus applied to the hand when the sound was administered within a limited distance from the hand. Moreover, results suggested that approaching sounds had a stronger spatially-dependent effect on tactile processing compared to receding sounds.

The ability to quickly localize stimuli on the body and in external space seems especially relevant in the context of pain. Indeed, potentially harmful objects approaching our body have to be quickly localized so that an appropriate defensive response can be prepared. In this study, we adapted the paradigm of Canzoneri et al. [18] to investigate the influence of dynamical *visual* stimuli on *nociceptive* processing. A visual stimulus was either approaching or receding the participant’s left or right hand. At different temporal delays from the onset of the visual stimulus, a nociceptive stimulus was applied either at the same or the opposite hand, so that it was presented when the visual stimulus was perceived at varying distances from the hand. Participants were asked to respond as fast as possible at which side they perceived a nociceptive stimulus. We expected that RTs to nociceptive stimuli would progressively decrease as a function of the perceived approach of the visual stimulus. Conversely, we expected RTs to increase as a function of the perceived recession of the visual stimulus. Moreover, we expected that this effect would be larger when visual stimuli were approaching/receding at the side of space in which the stimulated hand resided as opposed to when they were approaching/receding at the opposite side of space. The best fitting curves of the RTs as a function of the perceived position of the visual stimuli in space were studied in order to compare the influence of approaching versus receding visual stimuli on nociceptive processing.

## 2. Methods

### 2.1. Participants

30 paid participants volunteered to take part. Three participants (2 males, 1 female) were excluded because they failed to feel the stimulation despite repeated displacement of the electrodes (see section 2.2.). The final sample consisted of 27 participants (26 females, all right handed) with a mean age of 21 years (ranging from 18 to 26 years). All of the participants had normal or corrected-to-normal vision. Recent neurological, psychiatric or chronic pain diseases and usual intake of psychotropic drugs were considered as exclusion criteria. The experimental procedure was approved by the ethics committee of the faculty of psychology and educational sciences of Ghent University (2014/46). All of the participants provided written informed consent prior to taking part in the study.

### 2.2. Stimuli and apparatus

The nociceptive stimuli were delivered by means of intra-epidermal electrical stimulation (IES) (DS7 Stimulator, Digitimer Ltd, UK), with stainless steel concentric bipolar electrodes (Nihon Kohden, Japan; [19]). The electrodes consisted of a needle cathode (length: 0.1 mm, Ø: 1.4 mm). By gently pressing the device against the participant’s skin, the needle electrode was inserted into the epidermis of the dorsum of the hand in the sensory territory of the superficial branch of the radial nerve. Using intra-epidermal stimulation at maximum twice the absolute detection threshold was shown to selectively activate the free nerve endings of the Aδ fibers [19–21]. The detection threshold was determined with single-pulse stimuli (0.5 ms square wave pulse) using a staircase procedure [22]. The detection threshold was established separately for each hand. Next, the stimulus intensity was set at twice the detection threshold. If necessary, the intensity of the stimuli was adjusted so that the stimuli delivered to each hand were perceived as being equally intense. During the course of the experiment, the stimuli consisted of trains of four consecutive 0.5 ms square-wave pulses separated by a 5-ms inter-pulse interval. Using a set of pain words from the Dutch McGill Pain questionnaire [23] the stimuli were described as pricking. After each experimental block, the participants were asked to estimate the intensity elicited by the nociceptive stimuli on a numeric graphic rating scale (10 cm) with the following labels selected from the Dutch version of the McGill pain questionnaire (Vanderiet at al., 1987): 0 = felt nothing, 2.5 = lightly intense, 5 = moderately intense, 7.5 = very intense, 10 = enormously intense). This scale was used to ensure that: (1) the stimuli were still perceived, and (2) the percept elicited by the IES delivered to each of the participant’s hands was still equivalent. If one of these two criteria was not met, the stimulus intensities were modified (with a maximum intensity of 0.50 mA). If this adaptation proved to be unsuccessful (i.e. if one of the criteria was still not met), the electrodes were displaced and the procedure was restarted.

The visual stimuli were presented by means of fourteen green light-emitting diodes (LEDs), and a red LED for fixation.

The participants sat on a chair in a dimly illuminated, sound-attenuated room, with their head position fixed in a chin rest. The height of the chin rest was individually adapted. Participants rested their arms on the table in front of them, and placed their hands, palm downward on the table. The distance between the participants’ hands and their trunk, as well as the distance between the participants’ index fingers was 40 cm. In total 14 LEDs were positioned at different distances from the hands. 7 LEDs were positioned in the left side of space, and 7 LEDs in the right side of space. At both sides, the first LED was positioned in between thumb and index finger, the next six LEDs were positioned on a straight line one in front of the other with 12 cm in between successive LEDs, so that the last LED was 72 cm in front of the first LED. On each trial, the LEDs on one side were successively illuminated, creating the illusion of a light coming closer towards the participant (if the first LED illuminated was the LED at a distance of 72 cm from the participants), or going further away from the participant (if the first LED illuminated was the LED in between thumb and index finger). Each LED was illuminated for 280 ms, so that the total dynamical visual stimulus had a duration of 1960 ms. A red fixation LED was positioned in between the LEDs in left and right space, 36 cm in front of the first LEDs. This fixation LED was illuminated at the beginning of each trial, and was turned off for 1s at the end of each trial.

### 2.3. Procedure

The experiment started by illuminating the LEDs one by one. Participants were asked to look at the fixation LED and to indicate verbally at which side of space a light was illuminated (i.e. “left” or “right”). This was done to ensure that participants could see all the LEDs. Next, participants completed a practice phase of 14 trials, in which they executed the experimental task. Participants had to achieve 90% correct performance in this practice phase in order to proceed with the experiment.

**Fig 1:**
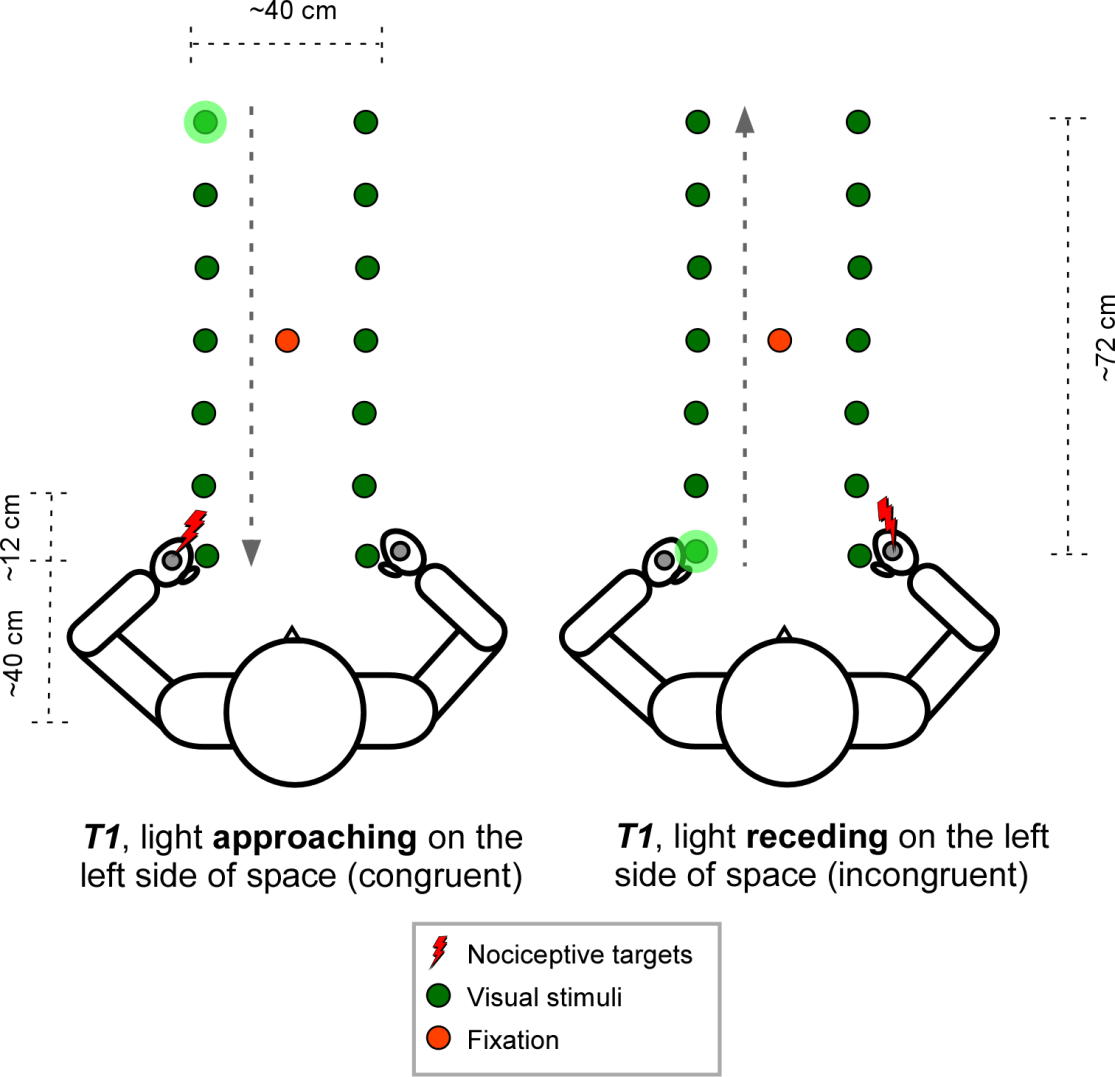
Experimental set-up. At the left side of the figure, a light is approaching the participant at the left side of space. At T1 (170 ms from light onset) the participant gets a nociceptive stimulation to the left hand (congruent to the side of space where the light is presented). At that time, the light is at 72 cm from the participants hand. At the right side of the figure, a similar situation is depicted, however now the light is receding from the participants hand, so that the light is in between the thumb and the index finger at the time of stimulation. Moreover, now the right hand is stimulated (incongruent to the side of space where the light is presented). The dashed arrow indicates the moving direction of the lights.

Each trial started with the illumination of the fixation LED for 1s. Thereafter the dynamical visual stimulus started. At different temporal delays after the onset of the visual stimulus, a nociceptive stimulus could be presented: T1, a nociceptive stimulus was administered 170 ms from light onset; T2, 450 ms from light onset; T3, 730 ms from light onset; T4, 1010 ms from light onset; T5, 1290 ms from light onset; T6, 1570 ms from light onset; T7, 1850 ms from light onset. This was true both for the approaching and the receding light. That way, the light was perceived at different locations with respect to the body at the moment the nociceptive stimuli were presented. For example, when the light was approaching it appeared close at high temporal delays. Conversely, when the light was receding, it appeared close at low temporal delays (see Fig 1).

The experiment consisted of 8 blocks of 56 trials each. The trials were created by crossing the moving direction of visual stimulus (approaching vs. receding) with the side at which the visual stimulus was presented (left vs. right side of space), the congruency of the visual and nociceptive stimulus (congruent vs. incongruent), and the 7 different temporal delays (T1—T7). 1/8 of the trials (i.e. 7 trials) per block were randomly assigned as catch trials, in which no nociceptive stimulus was presented.

Participants were instructed to keep their gaze on the fixation LED during the whole block. They were asked to respond as fast and accurately as possible which hand was stimulated (left or right hand). Responses were given by means of two foot pedals, one positioned beneath the toes, and one beneath their heel. Participants were instructed to keep the foot pedals depressed during the experiment, and to lift either their toes or their heel to respond. Participants were informed that the visual stimulus was unpredictive of the delivery of the subsequent nociceptive target. The experiment took on average 60 minutes to complete.

### 2.4. Measures

Because participants were highly accurate in performing the task (see section 3.3.), performance was only analyzed in terms of the reaction time (RT). Only RTs from correct trials were considered for analysis. RTs exceeding three times the median absolute deviation (MAD) [24] were considered outliers and were trimmed from the analyses (4% of trials on average over all conditions). Mean RTs were calculated for every temporal delay, for congruent and incongruent trials, and for approaching and receding visual stimuli, creating 28 different conditions.

After the experiment participants were asked to indicate how threatening they thought the visual lights were both when the light was approaching, and when the light was receding, on a scale from 0 (not at all) to 10 (extremely). The perceived threat score was compared for approaching and receding visual stimuli.

### 2.5. Analyses

Between each block participants were asked to rate the intensity of the stimulation for the left and the right hand on a numeric graphic rating scale (10 cm) with the following labels selected from the Dutch version of the McGill pain questionnaire [23]: 0 = felt nothing, 2.5 = lightly intense, 5 = moderately intense, 7.5 = very intense, 10 = enormously intense. The equivalence of the average current intensity and the average self-reported intensity for the left compared to the right hand was assessed using paired samples t-tests.

The perceived threat score was compared for approaching and receding lights using paired samples t-tests.

Mean accuracies were investigated to check whether any participants performed poorly on the task and therefore had to be excluded. However, accuracies were not of primary interest here, and were therefore not further analyzed.

The reaction time data was analyzed with R software [25] using linear mixed effects models as implemented in the package “lmerTest: tests in linear mixed effect models” [26,27]. Linear mixed effects models account for the correlations in within-subject data by estimating subject-specific deviations (or random effects) from each population-level factor (or fixed factor) of interest (see [28], for an elaboration). The outcome variable of interest was the RT. First all manipulated variables were taken into account, including the side of the stimulation (left versus right hand). However, as this variable did not interact with any of the other variables, it was left out of further analyses to increase power and for the sake of parsimony (see section 3.1.). The independent variables considered in the analysis were the *visual stimulus direction* (approaching vs. receding lights), the *congruency* of the nociceptive target (congruent vs. incongruent to the visual stimulus), and the *temporal delay* (T1 to T7). These were manipulated within subjects. Each analysis required three steps. First, all relevant factors and interactions were entered in the model as fixed factors, and we assessed whether it was necessary to add a random effect for each of the fixed factors in the analysis: If a random effect significantly increased the fit of the model, it was included in the final model. By default, a random effect was added introducing adjustments to the intercept conditional on the *Subject* variable. In the second step, we searched for the most parsimonious model that fitted the data. To achieve this, we systematically restricted the full model, comparing the goodness of fit using likelihood ratio tests. Finally, in the third step, we inspected the ANOVA table of the final model and tested specific hypotheses about possible main effects or interactions (for a similar approach, see [29–32]). P-values were calculated based on Satterthwaite’s approximations [33]. When an interaction effect was significant, it was further investigated with follow-up contrast analyses. The different steps in the model building procedure are illustrated in the supplementary information (S1 File).

## 3. Results

### 3.1. Intensity of the nociceptive stimulation

The mean current intensities used during the experiment were not significantly different for the left (*M* = 0.43 mA, *SD* = 0.05) and the right (*M* = 0.43 mA, *SD* = 0.07) hand, t(26) = 0.42, *p* = 0.68. These values correspond to those used in previous studies that succeeded to selectively activate nociceptors [19,20,34], and are much lower than those used in studies that failed to show selective activation [35].

However, the mean self-reported intensities (numeric graphic rating scale) were significantly lower for the left (*M* = 2.63, *SD* = 1.50) than for the right (*M* = 3.72, *SD* = 1.77) hand, t(26) = -3.54, p = 0.002. To check whether this difference in self-reported intensities had an effect on task performance, the *side of the nociceptive stimulus* was added to the model as additional variable. Although the main effect of *side* (F(1,9394.6) = 65.67; *p* < 0.001) was significant, indicating slower RTs when the left, compared to the right hand was stimulated, none of the interaction effects of *side* with any of the other variables (all F < 3.5; *p* > 0.05) were significant. For the sake of parsimony and to increase power, this variable was left out of further analyses.

In a number of trials participants didn’t feel anything, despite the fact that a stimulation to one of both hands was applied. On average 1% (±3%) of the stimuli was not felt. Two participants did not feel respectively 7% and 12% of the stimuli. However, these participants still had more than 80% correct responses in total, and were thus kept in the analyses (see section 3.3.).

### 3.2. Perceived threat value visual stimuli

Mean perceived threat scores were overall low, but significantly higher when the lights were approaching (*M* = 1.78, *SD* = 2.47) the participants, than when they were receding (*M* = 0.81, *SD* = 1.44), t(26) = 3.22, *p* = 0.003.

### 3.3. Accuracy

All participants had on average more than 80% correct task performance, and we decided to keep all participants in the analyses. Mean accuracy was 96% (± 4%). Accuracies were not further analyzed.

### 3.4. Reaction times

**Fig 2:**
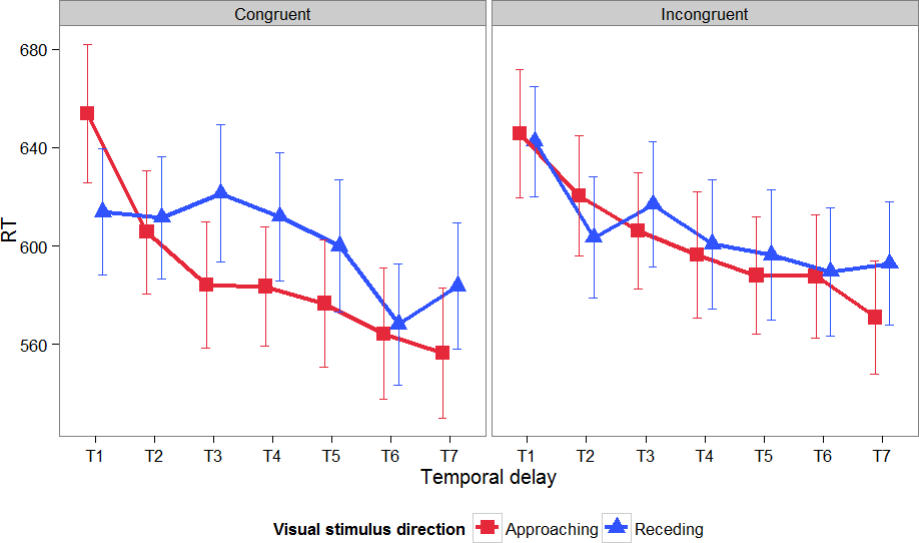
Mean RTs to the nociceptive targets and their associated standard errors in function of the different temporal delays at which the nociceptive stimuli were administered (from T1 to T7), the direction of the visual stimulus (approaching vs. receding) and the congruency of the nociceptive stimulation (congruent vs. incongruent to the visual cue).

The relationship between the RTs to the nociceptive targets, the different *temporal delays* at which the nociceptive stimuli were administered (from T1 to T7), the *visual stimulus direction* (approaching vs. receding) and the *congruency* of the nociceptive stimulation (congruent vs. incongruent to the visual cue) are represented in Fig 2.

The linear mixed effects model that demonstrated the best fit with the data, included all fixed factors together with their two-and three-way interactions, a random subject-based intercept, a random trial-based intercept and a random effect for *temporal delay* and *congruency*. In this final model, there was a significant main effect of *visual stimulus direction* (F(1,9414) = 12.04; *p* < 0.001), a significant main effect of *temporal delay* (F(6,30.8) = 12.21; *p* < 0.001), and a significant main effect of *congruency* (F(1,27.7) = 7.72; *p* = 0.01). Furthermore, the interaction effect between *visual stimulus direction* and *temporal delay* (F(6,9413.9) = 8.95; *p* < 0.001) and the three-way interaction between *visual stimulus direction*, *congruency*, and *temporal delay* (F(6,9398.4) = 3.76; *p* < 0.001) were significant. The interaction effect between *visual stimulus direction* and *congruency* (F(1,9381.7) = 2.30; *p* = 0.13) and between *congruency* and *temporal delay* (F(6,9385.5) = 1.51; *p* = 0.17) were not significant.

To further investigate the three-way interaction, two separate linear mixed effects models were fitted for congruent and incongruent trials with *visual stimulus direction* and *temporal delay* as independent variables and RT as dependent variable.

For *congruent* trials, the model that demonstrated the best fit with the data included the fixed factors and their interaction, a random subject-based intercept, a random trial-based intercept and a random effect for *temporal delay*. In this model, there was a main effect of *visual stimulus direction* (F(1,4642.7) = 11.85; *p* < 0.001), a main effect of *temporal delay* (F(6,29.7) = 14.88; *p* < 0.001), and an interaction effect between *visual stimulus direction* and *temporal delay* (F(6,4634.4) = 10.48; *p* < 0.001). Follow-up tests indicated that at T1, RTs were significantly slower for approaching than for receding visual stimuli (χ^2^(1) = 27.03, *p* < 0.001). This effect reversed at T3, T4, T5 and T7, where reaction times were significantly slower for receding than for approaching visual stimuli (T3: χ^2^(1) = 19.14, *p* < 0.001; T4: χ^2^(1) = 10.49, *p* = 0.001; T5: χ^2^(1) = 9.77, *p* = 0.002; T7: χ^2^(1) = 7.72, *p* = 0.005). At T2 and T6 reaction times did not differ significantly between approaching versus receding visual stimuli (T2: χ^2^(1) = 0.03, *p* = 0.86; T6: χ^2^(1) = 0.42, *p* = 0.52).

For *incongruent* trials, the final model consisted of all fixed factors, and their interaction, a random subject-based intercept, and a random effect for *visual stimulus direction* and *temporal delay*. In this model there was a main effect of *temporal delay* (F(6,28.1) = 8.32; *p* < 0.001), and a significant interaction effect between *visual stimulus direction and temporal delay* (F(6,4646.1) = 2.39; *p* = 0.03). The main effect of *visual stimulus direction* was not significant (F(1,27.3) = 1.14; *p* = 0.30). Follow-up tests indicated that at T2, RTs were marginally significantly faster for receding than for approaching trials (χ^2^(1) = 3.28, *p* = 0.07). Conversely, at T7, RTs were significantly faster for approaching than for receding trials (χ^2^(1) = 7.15, *p* = 0.008). None of the other comparisons were significant (all χ^2^ < 1.6; all *p* > 0.20).

Because the difference between receding and approaching trials for incongruent trials was only present at two time points and thus proved to be less consistent, further analyses focused on congruent trials. Pairwise comparisons between the different temporal delays for approaching visual stimuli showed that RTs at T1 were significantly slower than at any other temporal delay (all |t| > 5.00; all *p* < 0.001); RTs at T2 were significantly slower than reaction times at T3 to T7 (all |t| > 1.5; all *p* < 0.05); RTs at T3 were significantly slower than RTs at T6 and T7 (all |t| > 1.5; *p* < 0.05); RTs at T4 were marginally significantly slower than RTs at T6 (t(26) = -1.61; *p* = 0.06) and significantly slower than RTs at T7 (t(26) = -2.54; *p* = 0.009); finally RTs at T5 were marginally significantly slower than at T7 (t(26) = -1.70; *p* = 0.05). This provides an indication that for approaching visual stimuli, reaction times overall decreased. Moreover, this decrease was stronger for small temporal delays than for larger temporal delays. For receding visual stimuli, RTs remained stable at small temporal delays, and only dropped at T6 and T7. This is shown by a significant difference between RTs at T1 to T4 versus RTs at T6 and T7 (all |t| > 2.00; all *p* < 0.006), while RTs in either group did not differ significantly from each other (all |t| < 1.5; all *p* > 0.05). RTs at T5 were somewhere in between the two groups, as RTs at T5 did not differ significantly from RTs at T1, T2, T4 and T7 (all |t| < 1.5; all *p* > 0.05), but participants reacted significantly faster at T5 than at T3 (t(26) = -1.84; *p* = 0.04), and significantly slower at T5 than at T6 (t(26) = -3.20; *p* = 0.002).

**Fig 3:**
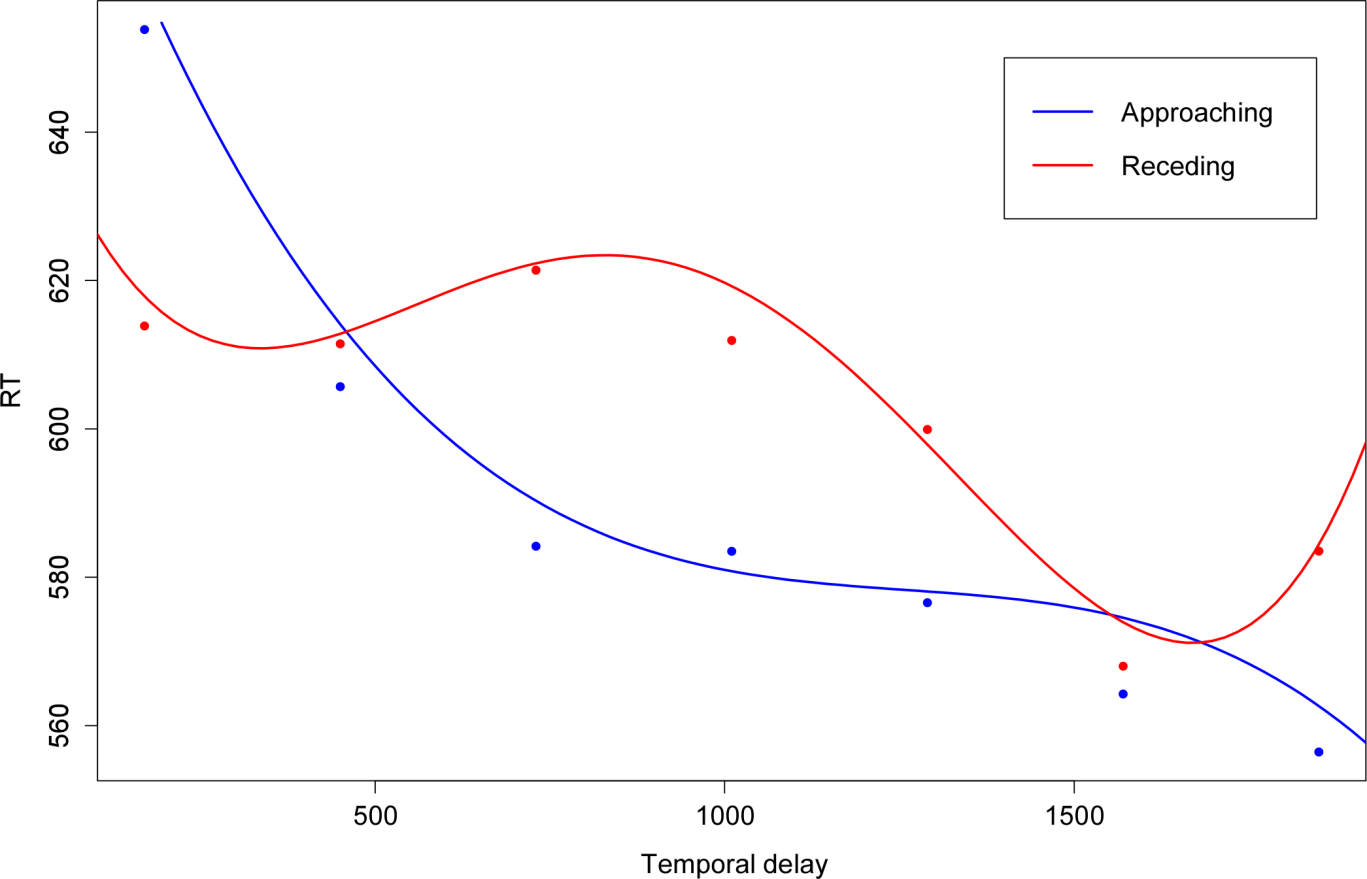
Mean RTs and fitted curves for the relationship between *temporal delay* and reaction time (RT) for congruent trials. For approaching visual stimuli a cubic model fitted the data best. For receding visual stimuli, a quartic model was used to describe the data.

Finally, we evaluated whether the model for congruent trials could be further simplified by considering *temporal delay* as a continuous variable instead of a factor, so that T1 corresponds to 170 ms, T2 to 450 ms, T3 to 730 ms, T4 to 1010 ms, T5 to 1290 ms, T6 to 1570 ms and T7 to 1850 ms. The nature of the relationship between the independent variable *temporal delay* and the dependent variable *RT* was investigated by fitting models with RT as dependent variable and *temporal delay* as independent variable separately for approaching and receding visual stimuli. At each time the restricted models (with *temporal delay* as continuous variable) were compared with the full model (with *temporal delay* as categorical variable). For approaching visual stimuli a linear relationship was first considered, assuming a constant decrease/increase of RT a as a function of temporal delay. This model fitted significantly worse than the model with *temporal delay* as a categorical predictor (χ^2^(5) = 35.30, *p* < 0.001). Next, a quadratic relationship was considered by adding the square of the independent variable *temporal delay* to the model. This model still fitted the data significantly worse than the full model (χ^2^(4) = 11.69, *p* = 0.02). Next, a cubic relationship was considered, and this model did not fit the data significantly worse than the full model (χ^2^(3) = 2.97, *p* = 0.40). For receding visual stimuli, the same strategy was applied. Again, the linear (χ^2^(5) = 19.79, *p* = 0.001) and the quadratic model (χ^2^(4) = 15.36, *p* = 0.004) fitted significantly poorer than the model with the categorical predictor. Now, also the cubic model fitted the data significantly worse (χ^2^(3) = 11.37, *p* = 0.01). Finally, a quartic model did not fit the data significantly worse (χ^2^(2) = 2.03; *p* = 0.36). The fitted curves are shown in Fig 3. The slopes of the tangent lines evaluated at each of the seven time points was calculated for the fitted curves for approaching and receding visual stimuli (see Table 1). For approaching visual stimuli, RTs decreased strongly at low temporal delays (T1 and T2), and remained more stable at higher temporal delays. For receding visual stimuli, RTs remain stable at low temporal delays (and even increased a little bit), to decrease only at higher temporal delays (from T5 onwards).

**Table 1:** Slopes of the tangent lines evaluated at the 7 time points.

	T1 (170 ms)	T2(450 ms)	T3(730 ms)	T4(1010 ms)	T5(1290 ms)	T6(1570 ms)	T7(1850 ms)
**Approaching**	-0.21	-0.12	-0.05	-0.02	-0.007	-0.023	-0.07
**Receding**	-0.09	0.03	0.02	-0.05	-0.10	-0.05	0.16

## 4. Discussion

This study investigated the influence of dynamical visual stimuli on nociceptive processing. Results showed that visual stimuli presented near the stimulated hand influenced nociceptive processing more than visual stimuli presented far from the hand, providing evidence for a body-part centered peripersonal frame of reference for the processing of nociceptive stimuli. Moreover, by using dynamical visual stimuli we were able to investigate the influence of visual stimuli along a continuous spatial range (from near to far space) both for approaching and receding stimuli.

To adequately defend ourselves against potential threats we need to be able to construct a coherent representation of our body and the space closely surrounding our body (i.e. the peripersonal space). Within this space the location of somatosensory stimuli, the location of visual stimuli close to the body and information about body posture are integrated [7,36,37]. In monkeys this ability depends on neurons with multimodal receptive fields (RFs), found mainly in the premotor and intraparietal areas [13,38]. These neurons are activated in response to both tactile stimuli and to visual stimuli occurring close to the stimulated body parts. In humans, the use of a peripersonal frame of reference for the localization of somatosensory stimuli has been demonstrated in neuropsychological studies with patients suffering from crossmodal extinction after a right hemisphere stroke. These patients can feel a tactile stimulation to their left hand in isolation, but when the right hand is concurrently stimulated (unimodal extinction) or when a right visual stimulus was presented near the right hand (crossmodal extinction) patients fail to report the left hand stimulation. However, when the right visual stimulus was presented far from the patients’ hand, the degree of extinction was reduced [39,40]. These results are in agreement with the electrophysiological findings from monkeys suggesting that the representation of peripersonal space is body-part centered [13]. Behavioral studies with healthy volunteers using a crossmodal congruency task [41–44] (for a review see [7]) found similar results.

Research investigating whether *nociceptive* stimuli are also mapped in a peripersonal frame of reference is more scarce. Dong et al. [6] found neurons in area 7b of monkeys that responded both to nociceptive stimuli and to visual stimuli approaching the receptive field of these neurons, especially when these visual stimuli were threatening or novel. Recently, we suggested the existence of a peripersonal frame of reference for mapping nociceptive stimuli in humans using temporal order judgment (TOJ) tasks [8,9]. In these tasks participants received two nociceptive stimulations, one to each hand, with different stimulus onset asynchronies (SOA’s) between both hands. Slightly before the first nociceptive stimulation a visual cue stimulus was presented either in the left or the right side of space, and either near or far from the participants’ hand. We found that visual stimuli presented near the stimulated hand facilitated processing of the nociceptive stimuli applied to that hand. Conversely, visual stimuli presented far from the hand only influenced nociceptive processing to a lesser extent [8,9]. In the current study we were able to replicate these findings showing that when the visual stimuli were presented at the side of space of the stimulated hand, reaction times at T1 were significantly faster for receding visual stimuli than for approaching visual stimuli. This can only be due to the fact that at this temporal delay, the visual stimulus was presented near the participants’ hand for receding visual stimuli, but far from the hand for approaching visual stimuli. This indicates that nociceptive processing was mostly facilitated when a visual stimulus was presented near as compared to far from the stimulated hand. This difference between approaching and receding visual stimuli at T1 was not significant when the visual stimuli were presented at the opposite side of space of the stimulated hand, indicating that it is especially the proximity to the stimulated body part and not so much to the body as a whole that is important. Taken together these results confirm previous findings with a different paradigm, and provide evidence for a peripersonal frame of reference centered on the stimulated body-part for the localization of nociceptive stimuli.

An important new aspect of the present study was the use of dynamical visual stimuli instead of static stimuli at two fixed positions (one near, one far) used in most previous studies. The use of moving stimuli is more ecologically valid and more comparable to animal studies investigating multimodal integration in the peripersonal space [5,6]. Furthermore studies in both humans and monkeys [13–17] have shown that the neural systems representing the peripersonal space show a preference for moving stimuli. By using dynamical visual stimuli, we were able to investigate multisensory integration along a continuum between near and far space. This was done by searching the best fitting function for the relationship between the RTs and the temporal delay at which the nociceptive stimuli were presented. This was only investigated for congruent trials, because the visual stimulus direction (approaching versus receding) most clearly affected the RTs for these trials, indicating that the distance of the visual stimuli to the body had a larger influence on RTs for congruent than for incongruent trials. For approaching trials a cubic function adequately described the data, indicating that RTs did not decrease linearly as a function of the approaching light. Indeed, the RTs dropped strongly in the beginning (T1 and T2), and decreased more slowly at higher temporal delays. This is also shown by the fact that RTs at low temporal delays (T1 and T2) were significantly higher than reaction times to nociceptive stimuli presented at higher temporal delays. For receding trials, a quartic function fitted the data well, indicating that reaction times did not increase/decrease linearly with the receding light. For these trials reaction times remained stable (and slightly increased) at low temporal delays, and then slowly decreased at higher temporal delays. It is surprising that despite the fact that the lights receded from the hand, reaction times nevertheless decreased at higher temporal delays (when the light was far away from the hand). Previous studies using a similar paradigm [18,45,46] also did not find the expected increase in RTs when stimuli were receding. However, in these studies RTs did not decrease at high temporal delays, but remained stable. It is important to note that there are some differences between these studies and the present study. First, these studies used auditory stimuli and tactile targets [18,45], or visual stimuli and tactile targets [46], instead of the visual stimuli and nociceptive targets used in the present study. Next, in the present study both the left or the right hand could be stimulated and the lights were approaching/receding at the same or the opposite side of space. Participants had to indicate which hand was stimulated (localization task). The previous studies only stimulated the right hand [18] or cheek [45] and participants had to indicate whether they felt a stimulation (detection task). Furthermore, Canzoneri et al. [18] and Serino et al. [46] also used ‘unimodal’ stimuli, i.e. tactile stimuli could occur during a silence period, preceding or following sound/visual stimulus administration. Serino et al. [46] used these unimodal trials as a baseline. Subtracting the fastest unimodal tactile condition from the bimodal conditions, gives a measure of the facilitation effect, due to the bimodal stimulation. They assessed the modulation of the facilitation effect in function of the temporal delay, instead of the raw RTs. An additional advantage of using unimodal trials is that it partly controls for spurious modulations of RTs due to an expectancy effect. Moreover, it controls for between-subject differences in RTs to tactile stimuli. Relatedly, Canzoneri et al. [18] and Teneggi et al. [45] had more catch trials (respectively 40% and ~33% out of the total amount of trials, compared to 12.5% in the present study). These catch trials should ensure that the expectation to receive a nociceptive stimulation to one of the hands does not increase with higher temporal delays. In the present study, catch trials were presented in 1/8 of the trials in each block. Given that no unimodal trials were used in the present experiment, it could be that the amount of catch trials was not sufficient to avoid the fact that people expected to get a stimulation, and that this expectation increased as the trial proceeded. We chose to eliminate the unimodal trials and to decrease the amount of catch trials to limit the overall amount of trials (and therefore the duration of the experiment) to ensure that participants could remain concentrated until the very end. These differences can be the cause of the decrease in RTs for receding stimuli. However despite this general effect of temporal delay, we were able to find a differential effect of visual stimulus direction (approaching vs. receding) on RTs, indicating that over and above the general decrease in reaction times with time, the direction of the lights significantly influenced RTs.

In accordance with the results of Canzoneri et al. [18] and Serino et al. [46] in the context of touch, our results suggest that the approaching lights had a stronger spatially dependent effect on nociceptive processing, compared to the receding lights. Indeed, the cubic function describing the relationship between RTs and the temporal delay at which nociceptive stimuli were delivered, showed a steep decrease immediately after the onset of the visual stimuli. Conversely, for the receding lights no such steep increase/decrease was present. In fact, reaction times remained stable and only decreased in the end, which is, as argued above, probably due to an increasing expectation of receiving a stimulation. These results are in agreement with studies in primates and humans showing adaptive avoidance responses to both real and simulated approaching stimuli [47–49]. For example, a rapidly expanding shadow elicits fear responses in rhesus monkeys [48] and human infants [50], but rapidly contracting shadows do not. Similarly, in the present study, participants rated the approaching stimuli as more threatening than the receding stimuli, albeit that the overall level of fear was low. Bimodal neurons in the ventral premotor cortex and the posterior parietal cortex of monkeys respond preferentially to approaching visual stimuli [51–53]. Moreover, Cooke and Graziano [4,54] found that when the monkeys’ brain regions that respond to approaching or nearby objects are stimulated, the animal executes defensive movements like withdrawing or blocking. At a behavioral level, humans process tactile stimuli applied to the cheek more rapidly when an object approached the cheek or the region closely surrounding the cheek, but not when this object was receding from the cheek [55]. These results can be explained by the fact that objects approaching us may pose a threat, and signal the need to initiate defensive behavior. Detecting these objects early is therefore crucial to either avoid the object, or prepare for contact most efficiently. In accordance with these results, Cléry et al. [56] demonstrated that tactile processing on the face can be enhanced by looming visual stimuli. More specifically, tactile processing was most enhanced when the tactile stimulus was applied at the expected time and location of impact of the looming visual stimulus. Therefore, the cortical network involved in the construction of the peripersonal space would play a key role in predicting the impact of a stimulus on our body [56]. Serino et al. [46] suggested that the degree of preference for approaching stimuli might vary for different body parts. These authors found that tactile detection on the hand was affected both by approaching and receding sounds, although receding stimuli had a less defined spatial gradient. Conversely, tactile detection applied to the trunk and the face was only affected by approaching sounds, and not by receding sounds. Moreover, comparing the boundaries of the peripersonal space around the hand, the face and the trunk, showed that the boundaries were smallest for the peri-hand space, intermediate for the peri-face space, and largest for the peri-trunk space. These findings are compatible with the function of the peripersonal space as a multisensory-motor interface for body-object interaction, either to plan an approaching movement, or to react to potential threats. Different body parts interact with objects over different portions of space: hand-object interactions occur within a limited space around the arm [57], face-object interactions mainly occur in the context of bringing an object to your mouth within the upper space [58], while trunk-object interactions materialize in a larger portion of space and are related to whole-body actions, such as walking [59]. Moreover, the hand usually receives touches both from approaching and receding stimuli, whereas it is much more likely that face or trunk tactile stimulation originates from an approaching stimulus. These studies suggest that the peripersonal frame of reference may constitute a safety margin around the body that is designed to protect it from potential physical threat and that represents a mechanism for preserving homeostatic control over the body [60,61]. Recently, it has been suggested that the peripersonal space representation cannot only be shaped by actions, but can also be modulated by emotional and social information (for a review, see [62]).

Neuroimaging studies have demonstrated that fronto-parietal brain regions, homologous to the brain regions hosting bimodal neurons in non-human primates, play an important role in the construction of a multimodal representation of the peripersonal space for tactile stimuli [17,63]. Based on the present study, it is reasonable to hypothesize that premotor and parietal areas also play an important role in nociceptive processing and pain perception [64]. Nociceptive inputs activate a large array of cortical areas, such as mainly opercular-insular and cingulated areas, but also frontal and parietal areas [65]. Recently, it was postulated that these areas are not specifically involved in nociceptive processing. Instead, activity in these areas would reflect the detection, localization and reaction to sensory events that are meaningful for the integrity of the body [64]. Based on the present and previous studies [8,9] it can be suggested that the involvement of frontal and parietal areas in nociceptive processing may serve the integration of nociceptive information into a multisensory representation of the body and the space closely surrounding the body.

This study has some limitations. First, the use of dynamical visual stimuli increased the ecological validity of this study. However, one could question the generalizability of a standardized experimental situation to real life. Indeed, it could be interesting to investigate the effect of real life objects (e.g. a syringe or a needle) approaching (or receding) from participants, as has been done in some animal studies (e.g. [6]) and recently also in humans [66,67]. For example, Rossetti et al. [67] investigated the skin conductance response (SCR) to a noxious stimulus (i.e. a needle) approaching and touching the hand, or stopping at different distances (near or far) from the hand. They found that anticipatory responses to an incoming threat were reduced when the stimulus targets a spatial position far away from the body, as compared to a near or bodily location. Despite the larger ecological validity of the use of real life objects, the use of standardized visual stimuli enabled us to investigate the influence of visual stimuli on nociceptive processing along a spatial continuum from near to far space, which would have been much more difficult to investigate in less standardized situations. Second, despite the procedure used to match the intensities of the nociceptive stimuli applied to both hands, the strict equivalence in subjective perception of the intensities between the two hands could not always be achieved. However, these differences were rather marginal (2.63 to 3.72 cm on a rating scale of 10 cm), and analyses showed that the side of stimulation did not affect the RTs. Finally, as mentioned above, we found a general effect of the temporal delay at which nociceptive stimuli were applied, which is most likely due to an increasing expectation to receive a nociceptive stimulus with time. Future studies could possibly avoid this by adding more trials without nociceptive stimulation (i.e. catch trials).

In conclusion, the present study provides evidence for the mapping of nociceptive stimuli in a peripersonal frame of reference. This guarantees a swift and efficient localization of threatening objects by integrating nociceptive information with visual information presented near the stimulated body part, enabling the preparation of a defensive motor response towards the location of threat. Moreover, by using dynamical visual stimuli we were able to investigate the relationship between nociceptive processing and the position of visual stimuli along a spatial continuum from near to far space. For approaching visual stimuli this relationship is best described by a cubic function, meaning that reaction times sharply decrease quickly after the onset of the visual stimulus. Conversely, for receding stimuli, no such sharp increase or decrease was found. This indicates that people are sensitive to the direction of visual stimuli, with approaching objects influencing nociceptive processing more profoundly than receding objects.

## Supporting Information

S1 File Model building procedure of linear mixed effect models. (DOCX)
