# Supplementary material for: What’s Coming Near? The Influence of Dynamical Visual Stimuli on Nociceptive Processing
Source: PLoS One. 2016 May 25;11(5):e0155864. doi: 10.1371/journal.pone.0155864 (PMC4880339; doi:10.1371/journal.pone.0155864)
Supplement: S1 File — (DOCX) [file pone.0155864.s001.docx]

**Supporting information – S1 File**

A top-down model building approach was adopted for fitting LME models (Pinheiro & Bates, 2000; West, Welch, & Galecki, 2007). First, a full model of the fixed effects was used to determine which random effects should be added to the model. The best pattern for the covariance of the residuals was chosen based on Restricted Maximum Likelihood estimation (REML estimation). Then, we determined which fixed effects needed to be included based on Maximum Likelihood estimation (ML estimation). Fixed effects were removed from the model if they didn’t significantly improve the fit of the model. Finally, the final model was refitted with REML estimation and the relevant contrasts were calculated. Below, the three fitting steps for each of the fitted models is presented. For each fitted model, Akaike’s information Criterion (AIC, Sakamoto, Ishiguro, & Kitagawa, 1986), the χ ^2^ for the relevant model comparisons, and the corresponding p-values are shown. The final table for each measure shows the Anova table, and the parameter estimates with their corresponding t-values.

**1. Model with visual stimulus direction, congruency and temporal delay (TD) as predictors.**

**Step 1. Full model. Determine random effects structure.**

| **Model** | Test | Random  Slope | Random Intercept | AIC | Df | χ ^2^ | | *p*-value | |
| --- | --- | --- | --- | --- | --- | --- | --- | --- | --- |
| **1** | Initial fit | 1 | Subject | 118190 | 30 |  | |  | |
| **2** | Random intercept trial number (1 vs. 2) | 1 | Subject, Trial | 118162 | 31 | χ ^2^(1) = 30.35 | < 0.001 | |  |
| **3** | Random slope visual stimulus direction (2 vs. 3) | 1 + visual stimulus direction | Subject, Trial | 118164 | 33 | χ ^2^(2) = 1.52 | 0.47 | |  |
| **4** | Random slope congruency (2 vs. 4) | 1 + congruency | Subject, Trial | 118152 | 33 | χ ^2^(2) = 13.61 | 0.001 | |  |
| **5** | Random slope TD  (4 vs. 5) | 1 + congruency + TD | Subject, Trial | 118078 | 66 | χ^2^(33) = 139.72 | <0.001 | |  |

Table 1. Decision: keep model 5 with random intercept for Subject and Trial number and random slope for congruency and temporal delay (TD).

**Step 2. Full model. Determine fixed effects.**

| **Model** | Test | Fixed | AIC | Df | χ ^2^ | *p*-value |
| --- | --- | --- | --- | --- | --- | --- |
| **1** | Initial fit | visual stimulus direction*congruency*TD | 118240 | 66 |  |  |
| **2** | Remove three-way interaction (1 vs. 2) | visual stimulus direction*congruency +  visual stimulus direction*  TD +  congruency*TD | 118251 | 60 | χ ^2^(6) = 22.52 | <0.001 |

Table 2. Decision: keep model 1 with the three-way interaction between visual stimulus direction, congruency and temporal delay (TD).

**Step 3. Test final model.**

| **Effects** | | **F** | **Df1** | | | **Df2** | | ***p*** | |
| --- | --- | --- | --- | --- | --- | --- | --- | --- | --- |
| **visual stimulus direction** | 12.04 | | | 1 | 9414.0 | | <0.001 | |  |
| **congruency** | 7.72 | | | 1 | 27.7 | | 0.01 | |  |
| **temporal delay (TD)** | 12.21 | | | 6 | 30.8 | | <0.001 | |  |
| **visual stimulus direction*congruency** | 2.30 | | | 1 | 9381.7 | | 0.13 | |  |
| **visual stimulus direction*TD** | 8.95 | | | 6 | 9413.9 | | <0.001 | |  |
| **congruency*TD** | 1.51 | | | 6 | 9385.5 | | 0.17 | |  |
| **visual stimulus direction*congruency* TD** | 3.76 | | | 6 | 9398.4 | | <0.001 | |  |
|  |  | | |  |  | |  | |  |
|  | ***B*** | | | **SE(B)** | **t** | | **CI** | |  |
| **Intercept** | 659.73 | | | 14.88 | 44.35 | | [630.57 to 688.89] | |  |
| **visual stimulus direction** | -44.95 | | | 8.49 | -5.29 | | [-61.60 to -28.30] | |  |
| **congruency** | -9.63 | | | 9.10 | -1.06 | | [-27.47 to 8.20] | |  |
| **temporal delay (T2)** | -49.01 | | | 9.91 | -4.95 | | [-68.44 to -29.59] | |  |
| **temporal delay (T3)** | -73.91 | | | 9.83 | -7.52 | | [-93.18 to -54.63] | |  |
| **temporal delay (T4)** | -73.84 | | | 10.47 | -7.05 | | [-94.37 to -53.31] | |  |
| **temporal delay (T5)** | -82.53 | | | 11.20 | -7.37 | | [-104.47 to -60.58] | |  |
| **temporal delay (T6)** | -95.30 | | | 10.93 | -8.72 | | [-116.73 to -73.87] | |  |
| **temporal delay (T7)** | -98.64 | | | 11.11 | -8.88 | | [-120.42 to -76.85] | |  |
| **visual stimulus direction*congruency** | 42.55 | | | 12.05 | 3.53 | | [18.94 to 66.16] | |  |
| **visual stimulus direction*T2** | 46.36 | | | 12.10 | 3.83 | | [22.65 to 70.07] | |  |
| **visual stimulus direction*T3** | 82.53 | | | 11.97 | 6.90 | | [59.07 to 105.98] | |  |
| **visual stimulus direction*T4** | 73.60 | | | 12.02 | 6.12 | | [50.04 to 97.16] | |  |
| **visual stimulus direction*T5** | 71.02 | | | 11.94 | 5.95 | | [47.61 to 94.43] | |  |
| **visual stimulus direction*T6** | 51.29 | | | 11.90 | 4.31 | | [27.96 to 74.62] | |  |
| **visual stimulus direction*T7** | 68.73 | | | 11.98 | 5.74 | | [45.26 to 92.21] | |  |
| **congruency*T2** | 22.70 | | | 12.17 | 1.87 | | [-1.15 to 46.55] | |  |
| **congruency*T3** | 35.86 | | | 12.04 | 2.98 | | [12.26 to 59.46] | |  |
| **congruency*T4** | 19.46 | | | 12.18 | 1.60 | | [-4.41 to 43.33] | |  |
| **congruency*T5** | 22.35 | | | 12.07 | 1.85 | | [-1.31 to 46.00] | |  |
| **congruency*T6** | 35.98 | | | 12.06 | 2.99 | | [12.35 to 59.61] | |  |
| **congruency*T7** | 23.37 | | | 12.10 | 1.93 | | [-0.33 to 47.08] | |  |
| **visual stimulus direction*congruency*T2** | -59.72 | | | 17.02 | -3.51 | | [-93.08 to -26.36] | |  |
| **visual stimulus direction*congruency*T3** | -70.66 | | | 16.92 | -4.18 | | [-103.82 to -37.50] | |  |
| **visual stimulus direction*congruency*T4** | -61.62 | | | 17.04 | -3.62 | | [-95.02 to -28.22] | |  |
| **visual stimulus direction*congruency*T5** | -58.55 | | | 16.90 | -3.46 | | [-91.68 to -25.42] | |  |
| **visual stimulus direction*congruency*T6** | -50.14 | | | 16.90 | -2.97 | | [-83.26 to -17.02] | |  |
| **visual stimulus direction*congruency*T7** | -45.16 | | | 17.00 | -2.66 | | [-78.48 to -11.85] | |  |

Table 3. Above: ANOVA table for the final model. Below: Parameter estimates (in ms) and their corresponding standard errors, t- values and confidence intervals.

**2. Model with visual stimulus direction and temporal delay (TD) as predictors.**

***2.1. Congruent trials***

**Step 1. Full model. Determine random effects structure.**

| **Model** | Test | Random  Slope | Random Intercept | AIC | Df | χ ^2^ | | *p*-value | |
| --- | --- | --- | --- | --- | --- | --- | --- | --- | --- |
| **1** | Initial fit | 1 | Subject | 59365 | 16 |  | |  | |
| **2** | Random intercept trial number (1 vs. 2) | 1 | Subject, Trial | 59353 | 17 | χ ^2^(1) = 14.19 | < 0.001 | |  |
| **3** | Random slope visual stimulus direction (2 vs. 3) | 1 + visual stimulus direction | Subject, Trial | 59355 | 19 | χ ^2^(2) = 1.37 | 0.50 | |  |
| **4** | Random slope TD (2 vs. 4) | 1 + TD | Subject, Trial | 59335 | 44 | χ ^2^(27) = 71.88 | <0.001 | |  |

Table 1. Decision: keep model 4 with random intercept for Subject and Trial number and random slope for temporal delay (TD).

**Step 2. Full model. Determine fixed effects.**

| **Model** | Test | Fixed | AIC | Df | χ ^2^ | *p*-value |
| --- | --- | --- | --- | --- | --- | --- |
| **1** | Initial fit | visual stimulus direction* TD | 59419 | 44 |  |  |
| **2** | Remove two-way interaction (1 vs. 2) | visual stimulus direction + TD | 59470 | 38 | χ ^2^(6) = 62.29 | <0.001 |

Table 2. Decision: keep model 1 with the two-way interaction between visual stimulus direction and temporal delay (TD).

**Step 3. Test final model.**

| **Effects** | | **F** | **Df1** | | | **Df2** | | ***p*** | |
| --- | --- | --- | --- | --- | --- | --- | --- | --- | --- |
| **visual stimulus direction** | 11.85 | | | 1 | 4642.7 | | <0.001 | |  |
| **temporal delay (TD)** | 14.88 | | | 6 | 29.7 | | <0.001 | |  |
| **visual stimulus direction*TD** | 10.48 | | | 6 | 4634.4 | | <0.001 | |  |
|  |  | | |  |  | |  | |  |
|  | ***B*** | | | **SE(B)** | **t** | | **CI** | |  |
| **Intercept** | 660.56 | | | 15.65 | 42.21 | | [629.88 to 691.23] | |  |
| **visual stimulus direction** | -45.20 | | | 8.70 | -5.20 | | [-62.25 to -28.16] | |  |
| **temporal delay (T2)** | -49.98 | | | 10.01 | -5.00 | | [-69.59 to -30.37] | |  |
| **temporal delay (T3)** | -74.64 | | | 11.07 | -6.74 | | [-96.34 to -52.95] | |  |
| **temporal delay (T4)** | -74.28 | | | 11.54 | -6.44 | | [-96.89 to -51.66] | |  |
| **temporal delay (T5)** | -83.89 | | | 11.95 | -7.02 | | [-107.32 to -60.47] | |  |
| **temporal delay (T6)** | -95.67 | | | 9.94 | -9.63 | | [-115.14 to -76.19] | |  |
| **temporal delay (T7)** | -99.49 | | | 11.58 | -8.59 | | [-122.18 to -76.79] | |  |
| **visual stimulus direction*T2** | 46.76 | | | 12.39 | 3.78 | | [22.48 to 71.04] | |  |
| **visual stimulus direction*T3** | 83.07 | | | 12.25 | 6.78 | | [59.07 to 107.08] | |  |
| **visual stimulus direction*T4** | 73.46 | | | 12.31 | 5.97 | | [49.33 to 97.58] | |  |
| **visual stimulus direction*T5** | 72.13 | | | 12.22 | 5.90 | | [48.17 to 96.09] | |  |
| **visual stimulus direction*T6** | 50.73 | | | 12.18 | 4.16 | | [26.85 to 74.61] | |  |
| **visual stimulus direction*T7** | 69.24 | | | 12.26 | 5.65 | | [45.21 to 93.27] | |  |

Table 3. Above: ANOVA table for the final model. Below: Parameter estimates (in ms) and their corresponding standard errors, t- values and confidence intervals.

***2.2. Incongruent trials***

**Step 1. Full model. Determine random effects structure.**

| **Model** | Test | Random  Slope | Random Intercept | AIC | Df | χ ^2^ | | *p*-value | |
| --- | --- | --- | --- | --- | --- | --- | --- | --- | --- |
| **1** | Initial fit | 1 | Subject | 58874 | 16 |  | |  | |
| **2** | Random intercept trial number (1 vs. 2) | 1 | Subject, Trial | 58874 | 17 | χ ^2^(1) = 2.05 | 0.15 | |  |
| **3** | Random slope visual stimulus direction (1 vs. 3) | 1 + visual stimulus direction | Subject | 58868 | 18 | χ ^2^(2) = 9.91 | 0.007 | |  |
| **4** | Random slope TD (3 vs. 4) | 1 + visual stimulus direction + TD | Subject | 58847 | 51 | χ ^2^(33) = 87.33 | <0.001 | |  |

Table 1. Decision: keep model 4 with random intercept for Subject and random slope for visual stimulus direction and temporal delay (TD).

**Step 2. Full model. Determine fixed effects.**

| **Model** | Test | Fixed | AIC | Df | χ ^2^ | *p*-value |
| --- | --- | --- | --- | --- | --- | --- |
| **1** | Initial fit | visual stimulus direction* TD | 58930 | 51 |  |  |
| **2** | Remove two-way interaction (1 vs. 2) | visual stimulus direction + TD | 58933 | 45 | χ ^2^(6) = 14.31 | 0.02 |

Table 2. Decision: keep model 1 with the two-way interaction between visual stimulus direction and temporal delay (TD).

**Step 3. Test final model.**

| **Effects** | | **F** | **Df1** | | | **Df2** | | ***p*** | |
| --- | --- | --- | --- | --- | --- | --- | --- | --- | --- |
| **visual stimulus direction** | 1.14 | | | 1 | 27.3 | | 0.30 | |  |
| **temporal delay (TD)** | 8.32 | | | 6 | 28.1 | | <0.001 | |  |
| **visual stimulus direction*TD** | 2.39 | | | 6 | 4646.1 | | 0.03 | |  |
|  |  | | |  |  | |  | |  |
|  | ***B*** | | | **SE(B)** | **t** | | **CI** | |  |
| **Intercept** | 648.63 | | | 14.12 | 45.94 | | [620.96 to 676.30] | |  |
| **visual stimulus direction** | -1.02 | | | 8.98 | -0.11 | | [-18.62 to 16.59] | |  |
| **temporal delay (T2)** | -24.90 | | | 10.99 | -2.27 | | [-46.44 to -3.36] | |  |
| **temporal delay (T3)** | -38.84 | | | 9.45 | -4.11 | | [-57.37 to -20.31] | |  |
| **temporal delay (T4)** | -53.13 | | | 10.62 | -5.00 | | [-73.94 to -32.32] | |  |
| **temporal delay (T5)** | -57.74 | | | 12.03 | -4.80 | | [-81.33 to -34.16] | |  |
| **temporal delay (T6)** | -56.85 | | | 12.12 | -4.69 | | [-80.59 to -33.10] | |  |
| **temporal delay (T7)** | -76.16 | | | 11.10 | -6.86 | | [-97.91 to -54.41] | |  |
| **visual stimulus direction*T2** | -14.86 | | | 11.55 | -1.29 | | [-37.50 to 7.79] | |  |
| **visual stimulus direction*T3** | 9.86 | | | 11.62 | 0.85 | | [-12.91 to 32.64] | |  |
| **visual stimulus direction*T4** | 12.08 | | | 11.72 | 1.03 | | [-10.90 to 35.05] | |  |
| **visual stimulus direction*T5** | 10.74 | | | 11.59 | 0.93 | | [-11.99 to 33.46] | |  |
| **visual stimulus direction*T6** | -0.89 | | | 11.63 | -0.08 | | [-23.69 to 21.91] | |  |
| **visual stimulus direction*T7** | 24.85 | | | 11.67 | 2.13 | | [1.99 to 47.72] | |  |

Table 3. Above: ANOVA table for the final model. Below: Parameter estimates (in ms) and their corresponding standard errors, t- values and confidence intervals.
